# ICTV Virus Taxonomy Profile: *Peribunyaviridae*

**DOI:** 10.1099/jgv.0.001365

**Published:** 2019-12-17

**Authors:** Holly R. Hughes, Scott Adkins, Sergey Alkhovskiy, Martin Beer, Carol Blair, Charles H. Calisher, Mike Drebot, Amy J. Lambert, William Marciel de Souza, Marco Marklewitz, Márcio R. T. Nunes, Xiǎohóng Shí (石晓宏), ICTV Report Consortium

**Affiliations:** ^1^​ Centers for Disease Control and Prevention, Fort Collins, CO, USA; ^2^​ United States Department of Agriculture, Agricultural Research Service, Fort Pierce, FL, USA; ^3^​ D. I. Ivanovsky Institute of Virology, Moscow, Russia; ^4^​ Friedrich-Loeffler-Institut, Greifswald-Insel Riems, Germany; ^5^​ Colorado State University, Fort Collins, CO, USA; ^6^​ Public Health Agency of Canada, Winnipeg, MB, Canada; ^7^​ University of Campinas (UNICAMP), Campinas, São Paulo, Brazil; ^8^​ Charité-Universitätsmedizin Berlin, Humboldt-University Berlin, and Berlin Institute of Health, Berlin, Germany; ^9^​ Evandro Chagas Institute, Ministry of Health, Ananindeua, Pará, Brazil; ^10^​ MRC-University of Glasgow Centre for Virus Research, Glasgow, UK

**Keywords:** *Peribunyaviridae*, bunyavirus, ICTV Report, *Orthobunyavirus*, *Herbevirus*, *Pacuvirus*, *Shangavirus*, taxonomy

## Abstract

Peribunyaviruses are enveloped and possess three distinct, single-stranded, negative-sense RNA segments comprising 11.2–12.5 kb in total. The family includes globally distributed viruses in the genera *Orthobunyavirus*, *Herbevirus*, *Pacuvirus* and *Shangavirus*. Most viruses are maintained in geographically-restricted vertebrate–arthropod transmission cycles that can include transovarial transmission from arthropod dam to offspring. Others are arthropod-specific. Arthropods can be persistently infected. Human infection occurs through blood feeding by an infected vector arthropod. Infections can result in a diversity of human and veterinary clinical outcomes in a strain-specific manner. Segment reassortment is evident between some peribunyaviruses. This is a summary of the International Committee on Taxonomy of Viruses (ICTV) Report on the taxonomy of the family *Peribunyaviridae*, which is available at ictv.global/report/peribunyaviridae.

## Virion

**Fig. 1.**
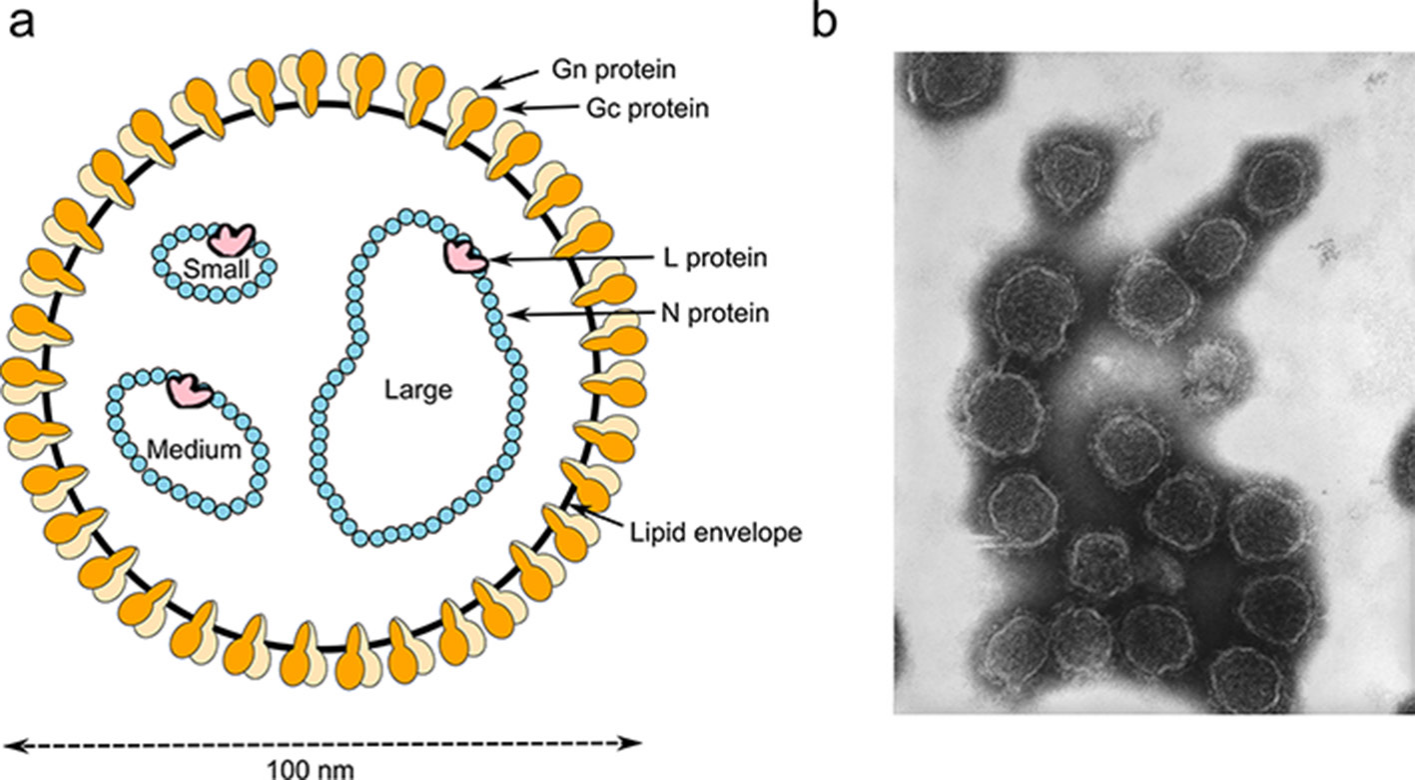
Peribunyavirus virion structure. (a) representation of a virion in cross-section. The surface spikes comprise the Gn and Gc glycoproteins. The helical nucleocapsids are circular and comprise one each of the unique ssRNA segments (L, large; M, medium; S, small) encapsidated by N protein and associated with the L protein. (b) negative-stained transmission electron microscopy photograph of California encephalitis virus virions (image: CDC/Drs Frederick Murphy and Erskine Palmer).

**Fig. 2.**
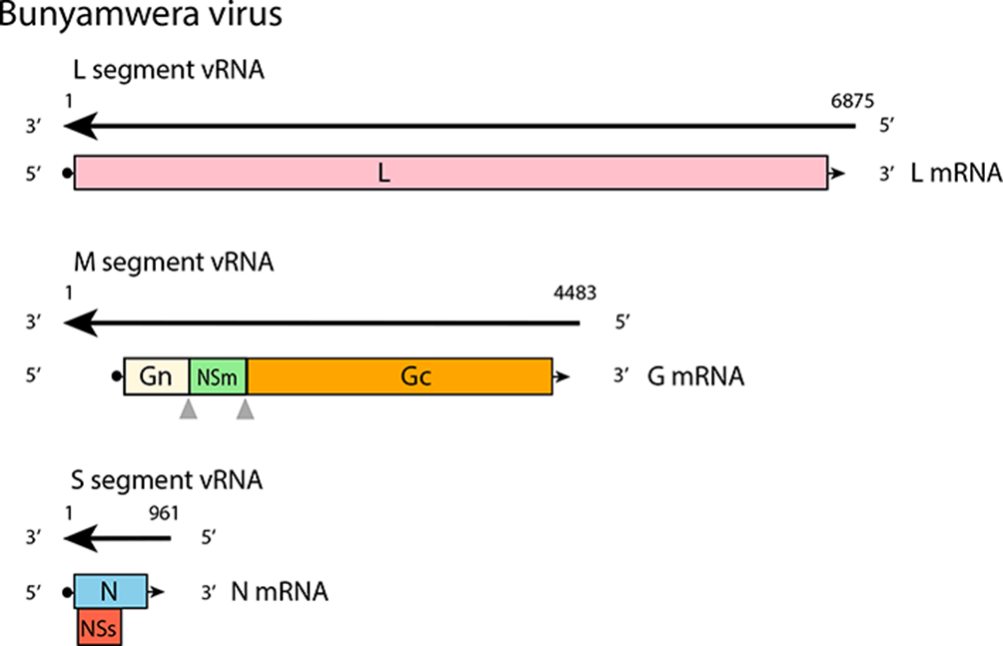
Coding strategy of Bunyamwera virus. Translation of NSs protein is initiated at an alternative start codon. The Gn, NSm and Gc proteins are generated by co-translational cleavage of M polyprotein.

Peribunyavirus virions are spherical or pleomorphic, 80–120 nm in diameter [1] with glycoprotein surface projections (5–18 nm) embedded in a lipid bilayer envelope (about 5 nm) (Table 1, Fig. 1). The genome comprises three single-stranded negative-sense RNAs (designated S, small, M, medium and L, large) (Fig. 2), each with complementary terminal nucleotide sequences that base-pair to form non-covalently closed, circular RNAs [2] that are individually encapsidated.

**Table 1.** Characteristics of members of the family *Peribunyaviridae*

Typical member	Bunyamwera virus (S, D00353; M, M11852; L, X14383), species *Bunyamwera orthobunyavirus*, genus *Orthobunyavirus*
Virion	Enveloped, spherical or pleomorphic virions, 80–120 nm in diameter
Genome	Three single-stranded, negative-sense RNA molecules, S, M and L, each of about 1, 4 and 6.8 kb
Replication	Cytoplasmic; primary transcription is primed by ‘cap snatching’ of host RNAs
Translation	On ER-bound ribosomes for Gn and Gc and on free ribosomes in the cytoplasm for N and L
Host range	Vertebrates and invertebrates (including mammals, birds, mosquitoes, culicoids and psychodid sandflies)
Taxonomy	Phylum *Negarnaviricota*, subphylum *Polyploviricotina*, class *Ellioviricetes*, order *Bunyavirales*, several genera and >90 species

## Genome

The S segment encodes the nucleocapsid protein (N), which is abundant in infected cells; in some viruses an overlapping reading frame encodes the non-structural protein NSs [3–5]. The M segment encodes two structural glycoproteins (Gn and Gc). Some members also encode a non-structural protein (NSm) between the Gn and Gc coding regions [4]. The L segment encodes the L protein, which has RNA-directed RNA polymerase and endonuclease functions.

## Replication

Virions attach via surface glycoproteins, entering the cell through clathrin-mediated endocytosis. Fusion of the viral Gc protein fusion peptide with endosomal membranes facilitates the release of ribonucleocapsids into the cytoplasm. The complementary 5′- and 3′-terminal ends serve as promoters for both mRNA and antigenome synthesis. Viral mRNAs are not polyadenylated and are truncated relative to the vRNA; a 5′-methylated cap is derived from host mRNA via ‘cap snatching’ mediated by the endonuclease function of the L protein. Proteins are translated on free ribosomes (S and L segment mRNAs) or membrane-bound ribosomes (M segment mRNA). The Gn and Gc proteins are generated by co-translational cleavage and targeted to and retained in the Golgi complex. Ribonucleoproteins are targeted near the Golgi complex. Genomes are packaged by signals from non-conserved sequences in the terminal untranslated regions. Virions bud into Golgi cisternae and are transported to the cell surface by the secretory pathway [6].

## Taxonomy

Genera are monophyletic based on analysis of the virus L protein; members of a genus have similar genomic organizations and transmission cycles. Peribunyaviruses form a group in the phylum *Negarnaviricota*, subphylum *Polyploviricotina*, class *Ellioviricetes*, order *Bunyavirales*﻿, being most closely related to viruses of the families *Fimoviridae* and *Tospoviridae*. Peribunyaviruses share some of the following characteristics: (i) enveloped spherical or pleomorphic virions; (ii) three segments of single-stranded, negative-sense RNA, with all proteins encoded in the same sense; (iii) capped but not polyadenylated viral mRNA; (iv) establish a persistent infection in an arthropod vector.

## Resources

Current ICTV Report on the family *Peribunyaviridae*: ictv.global/report/peribunyaviridae.
